# Validation study of the Amharic version Safety Attitudes Questionnaire (SAQ) in public hospitals of Addis Ababa, Ethiopia: a cross-sectional study

**DOI:** 10.1186/s12913-024-10865-9

**Published:** 2024-03-22

**Authors:** Bisrat Tamene Bekele, Trhas Tadesse Berhe, Biniam Yohannes Wotango, Wubet Mihretu Workneh, Nebiyou Wendwessen

**Affiliations:** 1 Addis Ababa City Administration Health Bureau, P.O. Box 316, Addis Ababa, Ethiopia; 2 Public Health Department, Yekatit 12 Hospital Medical College, Addis Ababa, Ethiopia; 3 Institute for Health Care Improvement, Addis Ababa, Ethiopia

**Keywords:** Safety attitude questionnaire, SAQ, Patient safety, Healthcare quality, Translation, Psychometric assessment, Amharic, Addis Ababa, Ethiopia

## Abstract

**Background:**

In Ethiopia, there is a growing concern about improving patients’ safety in healthcare facilities. However, the lack of a valid and reliable instrument sensitive to the Ethiopian culture for measuring health professional practice environment leads to difficulty in constructing evaluations of safety climate and further linking organizational research to outcomes research. This research study examined the psychometric properties of the Safety Attitude Questionnaire (SAQ) in the Amharic language within an Ethiopian healthcare context.

**Method:**

A hospital-based cross-sectional study design was conducted. The SAQ was meticulously translated into Amharic using forward and backward translation methods. Content validity was evaluated with input from seven patient safety and healthcare quality experts. Face validity was established through feedback from healthcare professionals. Then, the Amharic SAQ (SAQ-A) was distributed to 648 participants working in 11 public hospitals, and a total of 611 valid questionnaires were completed and returned (95.2% response rate). Cronbach’s alpha, McDonald’s omega, composite reliability, correlation analysis, and average variance estimation were calculated, and confirmatory factor analysis was performed. Descriptive analyses were performed to describe socio-demographic characteristics. A *P*-value of ≤0.05 was considered statistically significant. Tables, figures, charts, and texts are used for data presentation.

**Result:**

The overall internal consistency (Cronbach’s alpha) for the 31-item SAQ-A was 0.903, indicating excellent reliability. Confirmatory factor analyses demonstrated a good model fit for each dimension and the entire construct (χ2=1086.675, df=412, *p*<0.001, comparative fit index (CFI)=0.923, Tucker Lewis index (TLI)=0.913, and root mean square error of approximation (RMSEA)=0.052). The positive response rate of healthcare workers in hospitals was 32.1%. The positive response rates of the six dimensions were teamwork climate (59.7%), safety climate (41.9%), job satisfaction (57.1%), working conditions (37.5%), perception of management (37.6%), and stress recognition (46.2%).

**Conclusion:**

The Amharic translation of the SAQ showed good psychometric properties, making it a valuable tool for assessing safety attitudes among Amharic-speaking Ethiopian healthcare practitioners.

**Supplementary Information:**

The online version contains supplementary material available at 10.1186/s12913-024-10865-9.

## Introduction

Safety culture is an aspect of organizational culture that refers to the views, perceptions, and actions of personnel within an organization toward safety management and policy [1]. According to the WHO, the safety culture of an organization is the result of individual and group values, attitudes, perceptions, competencies, and patterns of behavior that determine an organization's commitment to, as well as the style and proficiency of, health and safety management [2]. Safety culture is an important core component of quality and safety improvement efforts. The Institute of Medicine (IOM) report Keeping Patients Safe emphasizes that within a strong culture of safety, health professionals will be more vigilant and report errors and near misses, thus nurturing a continuous learning environment [3].

Safety culture is a reliable predictor of clinical safety behaviors and patient safety outcomes. Improved safety culture has been shown to improve providers' psychological health as well as their engagement and satisfaction at work. Ultimately, a healthier culture benefits both patients and providers [4]. Several studies have shown that a positive safety culture can promote safe behavior among medical personnel, reduce the occurrence of adverse medical events, reduce the patient readmission rate, and decrease hospitalization time [5, 6].

In Ethiopia, there is a growing concern about improving patient safety in healthcare facilities [7]. Few studies have addressed the topic, while their findings are similar to the international literature [8–10]. A common barrier to assessing patient safety culture is the lack of a reliable and valid Amharic translation of instruments designed to assess various dimensions of patient safety and link those findings to outcomes.

The safety attitude questionnaire (SAQ) is the most widely used instrument for measuring patient safety culture [11]. Sexton et al. created the SAQ at the University of Texas [12]. The SAQ evolved from the Intensive Care Management Attitudes Questionnaire, which was refined from the Flight Management Attitudes Questionnaire used in the aviation industry (FMAQ). The survey was initially tested for psychometric properties in 203 different clinical settings across three countries (the United States, the United Kingdom, and New Zealand), with a total of 10,843 respondents. The questionnaire has been tailored to specific clinical areas [12]. The SAQ is a valid, reliable and psychometrically sound instrument and has been demonstrated to be responsive to interventions [12–14]. Sexton et al. demonstrated in their study that SAQ scale reliability is reported to be strong at 0.90 (Raykov’s ρ coefficient). A rigorous six-factor multilevel confirmatory factor analysis revealed a highly satisfying set of goodness-of-fit indicators: *p*<0.0001, comparative fit index (CFI) = 0.90, root mean square error of approximation (RMSEA) = 0.03, standard root mean square residuals (SRMSR) = 0.17 (between clinical areas) and SRMSR = 0.04 (within clinical areas) [12].

The aim of this study was to adapt the SAQ for use in Amharic speaking Ethiopian hospitals, assess the psychometric properties of the tool and present benchmarking data. Our research hypotheses related to the study aim were as follows:

- Hypotheses related to translation and content validity:
  - **Hypothesis 1 (H1): **The translation process from English to Amharic results in a culturally and linguistically equivalent version of the SAQ, as determined by expert evaluations and back-translation.
  - **Hypothesis 2 (H2): **The content validity assessment of the Amharic SAQ, as conducted by patient safety and healthcare quality experts, yield a satisfactory content validity index (CVI),
- Hypotheses related to reliability and construct validity:
  - **Hypothesis 3 (H3):** The translated Amharic SAQ demonstrates acceptable internal consistency, as measured by Cronbach's alpha, MacDonald’s omega coefficient, and Composite reliability.
  - **Hypothesis 4 (H4): **The construct validity of the Amharic SAQ is supported by confirmatory factor analysis (CFA), revealing a factor structure consistent with the original English version,

## Methods

### Study design, setting and period

The study was conducted in public hospitals in Addis Ababa, Ethiopia. A hospital-based cross-sectional, methodological design based on cross-cultural research translation theory, measurement theory, and psychometric theory [15] was constructed to translate and evaluate the psychometric properties of the Ethiopian Amharic Version Safety Attitude Questionnaire from 15 March 2023 to 15 April 2023 in Public Hospitals of Addis Ababa, Ethiopia.

Eleven hospitals in Addis Ababa participated in the study: eight city administration hospitals and three university hospitals. These hospitals were of variable size; the smallest covered was a maternal and child health hospital with 92 beds and the largest was a tertiary level teaching hospital which had 820 beds. There are a total of 11,273 health workers working in these facilities.

### Participants

There was no general agreement among researchers to determine the sample size for factor analysis. Additionally, there is no shortage of recommendations regarding the appropriate sample size to use to conduct factor analysis. Some authors recommend using a criterion based on the total sample size ranging from 100 to over 1000 [16, 17]. Others have suggested minimums for sample size based on a ratio of the number of cases to the number of variables involved in the factor analysis, including from 3 to 20 times the number of variables [16, 18]. Based on the above suggestions, a total of 648 eligible health professionals were invited for particiaption.

#### Instruments

##### Sociodemographic and occupation questionnaire

A demographic and occupation questionnaire collected background information regarding the participant. Information included the participant’s working hospital, age, sex, educational background, years of professional practice, average monthly income, profession, working department, and service area.

##### Safety attitude questionnaire

The Safety Attitudes Questionnaire was developed by Bryan Sexton, Eric Thomas, and Bob Helmerich with funding from the Robert Wood Johnson Foundation and Agency for Healthcare Research and Quality (https://med.uth.edu/chqs/surveys/safety-attitudes-and-safety-climate-questionnaire) [12]. The SAQ 25% of the items are from the Flight Management Attitude Questionnaire (FMAQ), and 75% of the items pertain to healthcare industry characteristics.

The generic SAQ short form version adopted for this study contains six safety-related dimensions: Teamwork Climate (6 items): perception of the quality of the collaboration among the team's professionals (Items 1-6); Safety Climate (7 items): Safety environment: perception of a strong and proactive organizational commitment to safety (Items 7-13); Job Satisfaction (5 items): pleasant feeling or emotionally positive state resulting from the perception of the work experience (Items 15-19); Stress Recognition (4 items): recognition of how the performance is influenced by stressful factors (Items 20-23); Perception of Management (4 items): approval of the management's actions regarding safety issues (Items 24-29). Each of these items is measured at two levels (perception of service management and perception of hospital management) and working conditions (5 items): perception of the quality of the environmental and logistical support within the workplace (e.g., equipment and professionals) (Items 30-32). Five items (items 14 and 33-36) are not part of the dimensions referred to above, and items 2, 11 and 36 are written in reverse. As such, the SAQ consists of a total of 41 items.

Each SAQ item is measured using a five-point Likert scale that ranges from strongly disagree to strongly agree with a neutral midpoint category (1 = Strongly Disagree, 2 = Slightly Disagree, 3 = Neutral, 4 = Slightly Agree, 5 = Strongly Agree). Items are scored and linearly transformed to a 0–100 scale (1 = 0, 2 = 25, 3 = 50, 4 = 75, 5 = 100). Higher scores on the SAQ reflect higher levels of safety culture.

### Translation and cultural adaptation

The corresponding author obtained permission to translate and use the SAQ short form from 2007 into the Amharic language through email from B Sexton, The University of Texas at Houston – Memorial Hermann Center for Healthcare Quality and Safety Houston, Texas, USA.

For the translation process the authors followed the survey instrument translation process where forward and backward translations followed by content and face validity assessments based on the “Systematic Survey Instrument Translation Process for Multi Country, Comparative Health Workforce Studies” recommendation [19].

The English version of the SAQ was translated into Amharic by two proficient independent bilingual translators (T1 and T2) with experience in instrument translation who produced two forward translations (FT1 and FT2). This resulted in two distinct forward-translated versions, which allowed for the initial exploration of potential linguistic and semantic variations. Then, an expert panel composed of the two translators, the principal investigator and an additional English and Amharic language expert reviewed the forward translations and reconciled them into one common forward translation (CFT) (Supplementary Table 1). A third bilingual translator who was unfamiliar with the original SAQ performed a blind back-translation (BT) of the CFT into English without access to the original source. The back translation aimed to ensure conceptual equivalence of the Amharic version to the original English SAQ. Finally, an expert committee comprising the translators, principal investigator, and a patient safety expert reviewed the CFT, BT and source to resolve any discrepancies and finalized the prefinal Amharic SAQ (SAQ-A) for expert review and pilot testing

Ambiguities and discrepancies regarding conceptual and semantic equivalence on three items were discussed and resolved by the committee members. Items that measured “Nurse input in the clinical area”, translated and modified into “  ” which means “Nurse or Midwife input in the clinical area”; “Morale of the clinical area” translated and modified into “  ”, which means “Self-confidence and work motivation of the clinical area” and “Trainees in my discipline” , was translated into “  ”, which means “trainees under my profession”.

### Content validity and face validity assessment and SAQ-A refinement

The content validity assessment was performed by a panel of seven patient safety and healthcare quality experts, each with extensive experience in healthcare settings and patient safety initiatives. The panel was diverse in terms of professional backgrounds and expertise. The assessment was performed and rated by the expert group with a two-way breakdown evaluation: Very relevant = 4; Somewhat relevant = 3; Weakly relevant = 2; Not related = 1. The content validity index at the item level (I-CVI) will be determined as part of the expert group according to the correlation of each item and the research concept. The acceptance level for CVI for the present study will be 0.80 and above.

The results of the content validity assessment demonstrated robust content validity for the Amharic SAQ (Supplemental Table 1). The CVI for relevance, which indicates the proportion of items rated as "quite relevant" or "highly relevant," was 0.97. Similarly, the CVI for clarity, reflecting the proportion of items rated as "item needs minor revision" or "item needs no revision", was 0.94. These high CVI scores indicate a consensus among the expert panel regarding the relevance and clarity of the SAQ items, affirming their content validity and surpassing the recommended threshold of 0.80, indicating strong content validity.

To gain a deeper understanding of the SAQ's content validity, the CVI was computed for each of its six scales: Teamwork Climate, Safety Climate, Job Satisfaction, Perceptions of Management, Working Conditions, and Stress Recognition. These subscale-level CVI scores consistently demonstrate the high relevance and clarity of the items within each subscale. The expert panel's consensus on the content validity of the subscales indicates their appropriateness for assessing safety attitudes in various healthcare settings.

The pilot testing phase of this study aimed to assess the initial reception and usability of the Safety Attitude Questionnaire (SAQ) among a diverse group of 18 participants. The pilot testing participants encompassed a heterogeneous sample, reflecting the multidisciplinary nature of healthcare settings: 4 (22.2%) Physicians, 4 (22.2%) nurses, 3 (16.7%) Public Health Officers, 3 (16.7%) Midwives, and 4 (22.2%) Pharmacy Professionals. This diverse representation ensured a comprehensive assessment of the SAQ across various healthcare roles. Participants were asked to evaluate the clarity and comprehensibility of each SAQ item on a 5-point Likert scale, ranging from 1 (not clear/not comprehensible) to 5 (very clear/very comprehensible). The overall ratings for item clarity were highly favorable, with a mean clarity rating of 4.29. Participants were also asked to provide feedback on the appropriateness of the SAQ items within the context of their roles and experiences. The overall ratings for item appropriateness were highly positive, with a mean appropriateness rating of 4.31. This indicates that, on average, participants considered the SAQ items to be appropriate for assessing safety attitudes within their respective healthcare roles.

#### Survey administration

In the data collection phase of our study, we employed Google Forms as the primary tool to gather responses from participants. Recognizing the importance of a smooth and efficient data collection process, we strategically engaged four Bachelor of Science in Nursing (BSC) professionals to facilitate and oversee the administration of the survey. These BSC nurses played a crucial role in maintaining a participant-friendly environment. They also provided clarifications on survey questions when needed and ensured that participants felt at ease throughout the process.

### Data processing and analysis

Given that data collection was conducted directly through Google Forms, traditional data entry procedures were not needed. Responses were automatically recorded in a digital format within the platform. However, data verification steps were undertaken to ensure the accuracy of data input in the online environment, such as reviewing the downloaded dataset for completeness and consistency. Then, the data were exported to STATA version 17 for further analysis. Psychometric analyses were performed to assess the fit of the expected factor structure and to test the validity and reliability of the Amharic version of the SAQ short form. To determine the factorability and adequacy of data for CFA, Kaiser‒Meyer‒Olkin (KMO) and anti-image correlation (MSA) were used, a value of more than 0.50 was accepted, and Bartlett's sphericity test (p<0.001) was performed to confirm patterns of the data. Several fit indices were examined to evaluate how well the models fit the observed data: RMSEA ≤ 0.06 (90% CI ≤ 0.06), SRMR ≤ 0.08, CFI ≥ 0.90, and TLI ≥ 0.90. Additionally, the chi-square/df ratio ≤ 3 rule was also used.

## Result

### Characteristics of participants

A total of 648 questionnaires were distributed, and 617 were collected, resulting in a significant response rate of 95.2%. Initial screening showed 6 questionnaires to be invalid, and these were therefore excluded, leaving a sample of 611. The mean age ± SD of the respondents was 34.05±7.19 years, and nearly half 288 (47.1%) were males. Regarding profession, the majority were nurses (234, 38.6%), followed by physicians (142, 23.4%). The demographic profile included various experience levels, with participants reporting an average of 8.34 ± 5.51 years of professional experience in their respective fields (Table 1).

**Table 1:** Sociodemographic characteristics of healthcare professionals

	**Characteristic**	**Frequency**	**Percent**
Age (years) (*N*=604)	Mean ± SD	34.04±7.18 years	
	20-29	177	29.3
	30-39	278	46.0
	40-49	131	21.7
	≥50	18	3.0
Sex (*N*=611)	Male	288	47.1
	Female	323	52.9
Educational status (*N*=611)	Degree/MD	455	74.5
	Master’s Degree/Specialty certificate	110	18.0
	Resident	46	7.5
Profession (*N*=607)	Nurse	234	38.6
	Physician	142	23.4
	Midwife	82	13.5
	Pharmacy professional	61	10.0
	Public health professional	49	8.1
	Laboratory technologist	31	5.1
	Social worker	8	1.3
Department (*N*=607)	Internal Medicine	127	20.9
	Gynecology	117	19.3
	Health care Quality	75	12.4
	Surgery	71	11.7
	Pediatrics	67	11.0
	Emergency and critical care	53	8.7
	Pharmacy	59	9.7
	Laboratory	30	4.9
	Social services	8	1.3
Unit (*N*=611)	Inpatient	212	34.7
	Outpatient	169	27.7
	Emergency	98	16.0
	ICU	44	7.2
	Quality/All	88	14.4
Experience in profession (years) (*N*=611)	Mean ± SD	8.34 ± 5.51 years	
	1-5	222	36.3
	6-10	200	32.7
	11-15	126	20.6
	>15	63	10.3

### Confirmatory factor analysis

#### Goodness-of-fit test

A structural equation model (SEM) was constructed to explore whether the index variable could be effectively used as a measure of the factors. The six-factor structure discovered by the literature review and the preliminary analysis was validated using a confirmatory factor analysis (CFA). The adequacy of the data for the implementation of this procedure was verified through the KMO (0.908) and the Bartlett test of sphericity (Χ^2^ (465) = 8989.453, *p* < 0.001), which revealed that the sample under study may be submitted to a CFA. The measurement models were evaluated using maximum likelihood estimation.

Based on the fit indices, the first CFA model exhibited a relatively poor fit. The modification indices revealed that certain items within the same factors may have correlation errors. This makes logical sense given that items measuring the same construct can have shared variance outside of what the factor can account for (Supplemental Figure 2).

The final CFA model incorporated two covariances between error terms and demonstrated improved fit (Table 2). The chi-square test remained significant (χ2=1086.675, df=412, *p*<0.001), which is expected with large samples. The incremental fit indices of CFI and TLI were 0.923 and 0.913, respectively, above the recommended 0.90 cutoff for acceptable fit. The RMSEA was 0.052, satisfying the preferred level below 0.06. Overall, the CFA provided confirmatory evidence that the six-factor model adequately fits the Amharic SAQ data. Additionally, SAQ-A showed that scale models generally fit the data well.

**Table 2:** Goodness-of-fit indices for CFA models of the Amharic SAQ

**Model**	**χ2**	**df**	**P**	**P close**	**GFI**	**CFI**	**TLI**	**RMSEA**	**SRMR**
**Initial**	**1217.906**	**429**	**<0.001**	**0.004**	**0.87**	**0.908**	**0.898**	**0.056**	**0.051**
**Final**	**1086.675**	**412**	**<0.001**	**0.209**	**0.88**	**0.923**	**0.913**	**0.052**	**0.049**
Teamwork climate	11.201	7	0.130	0.799	0.99	0.997	0.993	0.031	0.017
Safety climate	38.611	13	<0.001	0.266	0.98	0.984	0.075	0.057	0.025
Job satisfaction	3.804	4	0.433	0.890	0.99	1.000	1.000	<0.001	0.008
Stress recognition	1.429	2	<0.001	0.826	0.99	1.000	1.002	<0.001	0.007
Perception of management	15.727	3	0.001	<0.001	0.98	0.985	0.949	0.083	0.023
Working conditions	1.647	2	0.439	0.797	0.99	1.000	1.001	<0.001	0.007

### Factor loading

According to the results of the confirmatory factor analysis, it was found that all items and factors showed factor loadings > 0.5 (Supplementary Table 2). As a result, all items and factors were practically significant and feasible for use in data collection.

### Convergent validity and discriminant validity

Using Pearson's correlation coefficient (r), the relationships between the six SAQ-A scales were tested to determine the construct validity. Five of the six scales had substantial positive correlations with one another, as predicted (*p* value < 0.001), supporting the scale's convergent validity. This suggests that the scales all evaluate similar safety climate elements. The strongest correlation, which suggests that the notions on those scales overlap, was found between the two climates of teamwork and safety (*r*=0.622, *p* < 0.001). The working environment and stress recognition had the lowest correlation (*r*=0.007, *p* < 0.001), indicating that they measure relatively different constructs (Table 3). There were weak but significant correlations found between teamwork climate and stress recognition (*r* = -0.081) and work environment (*r* = 0.352) in the expected directions.

**Table 3:** Mean factor scores and intercorrelations between Amharic SAQ subscales

	Teamwork climate	Safety climate	Job Satisfaction	Stress Recognition	Management perception	Work Environment	Overall SAQ
Teamwork climate	1						
Safety climate	0.622^**^	1					
Job Satisfaction	0.467^**^	0.569^**^	1				
Stress Recognition	-0.081^*^	-0.038	-0.039	1			
Management perception	0.270^**^	0.457^**^	0.419^**^	0.047	1		
Work Environment	0.352^**^	0.382^**^	0.365^**^	0.007	0.416^**^	1	
Overall SAQ	0.663^**^	0.767^**^	0.733^**^	0.267^**^	0.674^**^	0.687^**^	1

^**^p<0.01, *p<0.05

The average variance extracted (AVE) was computed for each SAQ-A scale to gauge the proportion of variance in the items that is explained by the underlying construct relative to measurement error. The results showed that the AVE values for the six constructs were 0.47, 0.47, 0.60, 0.54, 0.44 and 0.59 for teamwork climate, safety climate, job satisfaction, stress recognition, perception of management and working conditions, respectively. As all constructs are nearly 0.5 and exceeded the threshold AVE value of >0.50, it is concluded that they could measure the latent variables. Hence, they fulfilled the convergent validity criteria. These six latent constructs had square roots of AVE: 0.68, 0.69, 0.78, 0.73, 0.65 and 0.77. The square roots of AVE of the six latent constructs were greater than the inter construct correlation. These results provide compelling evidence of discriminant validity, affirming that each SAQ-A construct is more strongly related to its own set of indicators than to those of other constructs (Table 4).

**Table 4:** Discriminant validity assessment for SAQ-A subscales

	Teamwork Climate	Safety Climate	Job Satisfaction	Stress Recognition	Management perception	Work Environment
Teamwork Climate	**0.679**					
Safety Climate	0.622	**0.685**				
Job Satisfaction	0.467	0.569	**0.777**			
Stress Recognition	-0.081	-0.038	-0.039	**0.733**		
Perception of Management	0.27	0.457	0.419	0.047	**0.644**	
Working Conditions	0.352	0.382	0.365	0.007	0.416	**0.765**

### Internal consistency

The internal consistency of the six factors and the 31 items of the translated Amharic version of the SAQ had composite reliability (CR) of 0.795 to 0.883 (Table 5). Job satisfaction had the highest CR score, and perception of management had the lowest value. The Cronbach’s alpha for the six scales ranged from 0.797 to 0.886, which were all considered acceptable reliability. Management perception had the lowest Cronbach’s alpha of 0.797, while job satisfaction had the highest value of 0.886. The overall Cronbach’s alpha the for the 31-item SAQ-A was 0.903. Additionally, McDonald's omega coefficients, which provide an alternative measure of internal consistency, mirrored these findings with values between 0.795 and 0.883 across subscales.

**Table 5:** Internal consistency reliability analysis of the Amharic SAQ

**Scale**	**No. of items**	**Cronbach's alpha**	**McDonald's omega**	**Composite reliability**
Teamwork climate	6	0.838	0.839	0.839
Safety climate	7	0.861	0.862	0.860
Job satisfaction	5	0.886	0.888	0.883
Stress recognition	4	0.820	0.822	0.822
Perceptions of management	4	0.797	0.797	0.795
Working conditions	5	0.846	0.852	0.849

#### Survey analysis

##### SAQ Score

The total mean score of the SAQ-A for the healthcare workers in eleven public hospitals in Addis Ababa was 68.13 ± 12.87 (Table 6). There was substantial variability ranging from 0% to 100% in the percent of positive scores for each of the factors. Working conditions and stress recognition showed the greatest variability, and teamwork climate showed the least variability. The positive response rate of the healthcare workers in the hospitals was 32.1%, which was 75% and above.

**Table 6:** SAQ-A subscale and total mean score

**Scale**	**Mean Score**	**SD**	**Positive response rate (%)**
Teamwork climate	74.82	17.54	59.7%
Safety climate	67.96	19.29	41.9%
Job satisfaction	71.41	21.98	57.1%
Stress recognition	66.88	22.65	46.2%
Perceptions of management	65.87	18.34	37.6%
Working conditions	61.86	23.66	37.5%
**Overall SAQ**	**68.13**	**12.87**	**32.1%**

The positive response rates of the six dimensions ranged from 37.5% to 59.7%. These rates were teamwork climate (59.7%), safety climate (41.9%), job satisfaction (57.1%), working conditions (37.5%), perception of management (37.6%), and stress recognition (46.2%).

## Discussion

This study aimed to translate the SAQ-A and evaluate its reliability and validity when used to assess patient safety culture in Ethiopian hospitals. The English SAQ was rigorously translated, incorporating back-translation and expert review. Comprehensive psychometric analysis provided evidence that the Amharic version demonstrates excellent reliability and validity mirroring the original questionnaire.

The SAQ-A shows good content validity at both the overall CVI of relevance of 0.97 and clarity of 0.94 and item level (I-CVI range: 0.71-1.00), according to expert assessment. This satisfies the newly constructed scale's recommended cutoff point of 0.80 [20]. Our scale-level content validity index aligned closely with the Arabic SAQ, which reported an S-CVI of 0.97 based on ratings by 10 experts [21]. In addition, similar rigorous interpretation and content validation procedures were performed in previous SAQ validation studies in China and Norway [22, 23]. This helps ensure language/cultural flexibility.

In this study, the CFA validated the six-factor structure and showed acceptable fit after adjusting the model. The factor structure mirrored the original English SAQ, which reported more than 60% of variance explained by six factors [12]. Our CFA findings were consistent with other SAQ validation studies that found acceptable model fit after allowing error term correlations [22, 24]. This likely reflects minor residual associations between items assessing the same underlying safety construct. In addition to the overall questionnaire CFA, the factor structure was tested individually for each of the six subscales. Every scale model showed strong goodness-of-fit based on the CFI, TLI, RMSEA, and standardized RMR fit statistics. This further confirms that the measurement model matches the hypothesized latent factors for each set of subscale items [22]. The robust CFA results align with previous studies consistently supporting the six-factor structure of the SAQ across translations in China, Portugal, and Switzerland [22, 25, 26].

The pattern of positive intercorrelations found between Amharic SAQ scales supported convergent validity. Conceptually, it was believed that there would be a substantial correlation between the climates for teamwork, safety, job satisfaction, management perception, and working circumstances. For instance, the teamwork climate and safety climate scales had the greatest correlation of.62, which was comparable with the initial validation in which those scales had a *r*=0.83 association [12]. This supports the idea that perceptions of teamwork and support have a favorable impact on feelings of safety norms. The weakest intercorrelation in our study was between stress recognition and working conditions (*r*=0.007), which are conceptually distinct domains. This aligns with the Chinese study, which reported that those subscales correlated poorly in their Chinese SAQ data [22]. The AVE for all six Amharic SAQ subscales was nearly 0.5 or exceeded 0.50, supporting convergent validity. Additionally, the AVE square roots were all greater than the inter scale correlations. This satisfied the Fornell-Larcker criterion that a scale shares more variance with its own items than with other constructs [27]. Together, these results provide evidence that while related, each safety climate dimension measured by the Amharic SAQ is distinct.

Alpha coefficients for the six subscales ranged from 0.79 to 0.89, all surpassing the widely accepted standard of 0.70 for reliable scales [28]. Safety climate had the highest scale reliability. Management perception had the lowest, although still adequate, reliability. These findings mirror the results of past SAQ validation studies that also found high internal reliability for the instrument. For example, the Arabic SAQ demonstrated Cronbach’s alphas between 0.747 and 0.822 for each subscale [21]. The Chinese SAQ administered in 208 hospitals had subscale alpha coefficients from 0.785 to 0.912 [29]. Similarly, a Polish version of SAQ has also shown a reliability of subscales ranging from 0.74 to 0.95 [30]. McDonald's omega, an alternative reliability measure, yielded equivalent values to alpha across subscales. Omega has advantages over alpha in being a more robust lower-bound estimate less sensitive to the number of items. The consistent alpha and omega values provide added evidence for the high internal reliability of the Amharic SAQ.

The average total SAQ score was 68.13, below the 75-point threshold considered a positive safety culture. This reveals considerable room for improvement in conditions and attitudes related to safety in Ethiopian hospitals. The highest scoring subdomain was teamwork climate, followed by job satisfaction and safety climate. The lowest scoring areas were working conditions and stress recognition. At the individual item level, the greatest concerns were around hospital training practices and staffing adequacy.

## Limitation

One of the limitations is sampling from only public hospitals limits representativeness of the nationwide healthcare workforce. Findings cannot be generalized to private facilities. In addition, unequal sample distribution with nurses and physicians outweighing other professionals could introduce bias. Caution needed when comparing differences across professions. The self-reported data may be vulnerable to social desirability bias affecting reliability. Cross-sectional design prevents assessment of predictive validity and responsiveness over time. Lastly, due to the limited scope of this study, it was not possible to assess factor structure invariance across age, sex, job categories, or other stratification variables of respondents.

## Conclusion

This study provided substantial evidence that the Amharic translation of the SAQ retains sound psychometric properties equivalent to the English version. The Amharic SAQ showed high internal consistency. Content validity was confirmed through expert reviews and pilot testing. Construct validity was established by the logical intercorrelations between subscales. The validated SAQ-A can be readily adopted to measure patient safety culture in Ethiopian hospitals. It allows objective assessment to identify strengths and weaknesses in safety climate, raising staff awareness and guiding data-driven quality improvements.

### Supplementary Information

**Supplementary Material 1.** 

**Supplementary Material 2.** 

**Supplementary Material 3.** 

## Data Availability

The datasets used and/or analyzed during the current study are available from the corresponding author on reasonable request.
