# Supplementary material for: Validation study of the Amharic version Safety Attitudes Questionnaire (SAQ) in public hospitals of Addis Ababa, Ethiopia: a cross-sectional study
Source: BMC Health Serv Res. 2024 Mar 22;24:366. doi: 10.1186/s12913-024-10865-9 (PMC10960426; doi:10.1186/s12913-024-10865-9)
Supplement: Supplementary file 1 — Supplementary Material 1. [file 12913_2024_10865_MOESM1_ESM.docx]

Supplementary table 1: SAQ translation and reconciliation

| **S.N.** | **Original English version** | **Amharic Translation 1** | **Amharic Translation 2** | **Reconciled Final Version** |
| --- | --- | --- | --- | --- |
|  | Nurse input is well received in this clinical area. | በምሰራበት የህክምና ክፍል የነርሶች ግብአት በደንብ ተቀባይነት ያለው፡፡. | በዚህ የህክምና ቦታ ላይ በነርስ የሚሰጥ አስተያየት ጥሩ ተቀባይነት አለው፡፡ | በዚህ የህክምና ክፍል ውስጥ በነርስ/በሚድዋይፍ የሚሰጥ አስተያየት ጥሩ ተቀባይነት አለው |
|  | In this clinical area, it is difficult to speak up if I perceive a problem with patient care. | በምሰራበት የህክምና ክፍል , በታካሚ እንክብካቤ ላይ ችግር እንዳለ ከተረዳሁ ለመናገር አስቸጋሪ ነው። | በዚህ የህክምና ቦታ ላይ የታካሚ እንክብካቤ ላይ ችግር አለ ብዬ ባስብ እንኳ ይህን ለመናገር አስቸጋሪ ነው፡፡ | በዚህ የህክምና ክፍል ውስጥ የታካሚ እንክብካቤ ላይ ችግር አለ ብዬ ባስብ እንኳ ይህን ለመናገር አስቸጋሪ ነው፡፡ |
|  | Disagreements in this clinical area are resolved appropriately (i.e., not who is right, but what is best for the patient). | በምሰራበት የህክምና አካባቢ ያሉ አለመግባባቶች በተገቢው መንገድ ይፈታሉ (ማለትም ማን ትክክል ነው በማለት ሳይሆን ለታካሚው የተሻለውን በመምረጥ) | በዚህ የህክምና ቦታ ላይ የሚፈጠሩ አለመግባባቶች በአግባቡ መፍትሄ ያገኛሉ (ይህም ማለት ትኩረት የሚደረገው *ማን ነው* ትክክል የሚለው ላይ ሳይሆን ለታካሚው የሚሻለው *ምንድን ነው* የሚለው ላይ ነው) | በዚህ የህክምና ክፍል ውስጥ የሚፈጠሩ አለመግባባቶች በአግባቡ መፍትሄ ያገኛሉ (ይህም ማለት ትኩረት የሚደረገው *ማን ነው* ትክክል የሚለው ላይ ሳይሆን ለታካሚው የሚሻለው *ምንድን ነው* የሚለው ላይ ነው) |
|  | I have the support I need from other personnel to care for patients. | ታካሚዎችን ለመንከባከብ ከሌሎች ሰራተኞች የምፈልገውን ድጋፍ አገኛለሁ | ለታካሚዎች እንክብካቤ በማድረግ ረገድ ከሌሎች ሰራተኞች የምፈልገውን ድጋፍ አገኛለሁ፡፡ | ታካሚዎችን ለመንከባከብ ከሌሎች ሰራተኞች የምፈልገውን ድጋፍ አገኛለሁ፡፡ |
|  | It is easy for personnel here to ask questions when there is something that they do not understand. | በምሰራበት የህክምና አካባቢ ላሉ ሰራተኞች ያልተረዱት ነገር ሲኖር ጥያቄዎችን መጠየቅ ቀላል ነው። | እዚህ ለሰራተኞች ያልተረዱት ነገር ሲኖር ጥያቄዎችን ለመጠየቅ ቀላል ነው፡፡ | እዚህ የህክምና ክፍል ውስጥ ያሉ ሰራተኞች ያልተረዱት ነገር ሲኖር ጥያቄዎችን ለመጠየቅ ቀላል ነው፡፡ |
|  | The physicians and nurses here work together as a well-coordinated team. | በምሰራበት የህክምና ያሉት ሐኪሞች እና ነርሶች በደንብ የተቀናጀ ቡድን ሆነው አብረው ይሰራሉ። | እዚህ የሚሰሩ ሀኪሞችና ነርሶች በጥሩ ሁኔታ እንደተቀናጀ ቡድን ሆነው አንድ ላይ ይሰራሉ፡፡ | በዚህ የህክምና ክፍል ውስጥ የሚሰሩ ሀኪሞችና ነርሶች እንደ አንድ ቡድን በደንብ ተቀናጀተዉ ይሰራሉ፡፡ |
|  | I would feel safe being treated here as a patient. | በምሰራበት የህክምና ክፍል እንደ ታካሚ ሆኜ ብታከም ደህንነት ይሰማኛል | እንደ ታካሚ እዚህ መታከም የደህንነት ስሜት ይሰጠኛል፡፡ | እንደ ታካሚ ሆኜ እዚህ ብታከም የደህንነት ስሜት ይሰጠኛል፡፡ |
|  | Medical errors are handled appropriately in this clinical area. | በምሰራበት የህክምና አካባቢ የሕክምና ስህተቶች በትክክል ይስተናገዳሉ | በዚህ የህክምና ቦታ ላይ የህክምና ስህተቶች በአግባቡ መፍትሄ ያገኛሉ፡፡ | በዚህ የህክምና ቦታ ላይ የህክምና ስህተቶች በአግባቡ መፍትሄ ያገኛሉ፡፡ |
|  | I know the proper channels to direct questions regarding patient safety in this clinical area. | በምሰራበት የህክምና አካባቢ የታካሚን ደህንነት በተመለከተ ጥያቄዎችን የማድረሻ ትክክለኛውን መንገድ አውቃለሁ | በዚህ የህክምና ቦታ ላይ የታካሚ ደህንነትን የተመለከቱ ጥያቄዎችን የምወስድባቸውን የግንኙነት መስመሮች አውቃቸዋለሁ፡፡ | በዚህ የህክምና ክፍል የታካሚን ደህንነት በተመለከተ ጥያቄዎችን የመጠየቂያ ትክክለኛውን መንገድ አውቃለሁ፡፡ |
|  | I receive appropriate feedback about my performance. | ስለ ስራ አፈፃፀሜ ተገቢውን ምላሽ አገኛለሁ | አፈጻጸሜን በተመለከተ ተገቢ ግብረ መልስ ይሰጠኛል፡፡ | የስራ አፈጻጸሜን በተመለከተ ተገቢ ግብረ መልስ (ምላሽ) ይሰጠኛል፡፡ |
|  | In this clinical area, it is difficult to discuss errors. | በምሰራበት የህክምና አካባቢ, ስህተቶችን ለመወያየት አስቸጋሪ ነው | በዚህ የህክምና ቦታ ላይ ስለ ስህተቶች መነጋገር ከባድ ነው፡፡ | በዚህ የህክምና ቦታ ላይ ስህተቶችን ለመወያየት አስቸጋሪ ነው፡፡ |
|  | I am encouraged by my colleagues to report any patient safety concerns I may have. | በታካሚ ደህንነት ላይ ሊያጋጥም የሚችል ማንኛውንም ስጋት እንዳሳውቅ ባልደረቦቼ ያበረታቱኛል | የስራ ባልደረቦቼ ሊኖሩኝ የሚችሉ ማንኛውንም የታካሚ ደህንነትን የተመለከቱ ስጋቶች ሪፖርት እንዳደርግ ያበረታቱኛል፡፡ | የስራ ባልደረቦቼ በታካሚ ደህንነት ላይ ሊያጋጥም የሚችል ማንኛውንም ስጋቶች ሪፖርት እንዳደርግ ያበረታቱኛል፡፡ |
|  | The culture in this clinical area makes it easy to learn from the errors of others. | በምሰራበት የህክምና አካባቢ ያለው ከስህተቶች የመማር ባህል ከሌሎች ስህተቶች ለመማር ቀላል ያደርገዋል | በዚህ የህክምና ቦታ ላይ ያለው የስራ ባህል ከሌሎች ስህተቶች መማርን ያቀላል፡፡ | በዚህ የህክምና ቦታ ላይ ያለው የስራ ባህል ከሌሎች ስህተቶች መማርን ያቀላል:: |
|  | I like my job. | ሥራዬን እወደዋለሁ | ስራዬን እወደዋለሁ፡፡ | ስራዬን እወደዋለሁ |
|  | Working here is like being part of a large family. | ስራ እዚህ መስራት የአንድ ትልቅ ቤተሰብ አባል የመሆን ያህል ነው። | እዚህ መስራት ልክ የትልቅ ቤተሰብ አባል የመሆን ያክል ነው፡፡ | እዚህ መስራት ልክ የትልቅ ቤተሰብ አባል የመሆን ያክል ነው፡፡ |
|  | This is a good place to work. | ይህ ለመስራት ጥሩ ቦታ ነው። | ይህ ለመስራት ጥሩ የስራ ቦታ ነው፡፡ | ይህ ለመስራት ጥሩ የስራ ቦታ ነው፡፡ |
|  | I am proud to work in this clinical area. | እዚህ የህክምና አካባቢ በመስራቴ ኩራት ይሰማኛል። | እዚህ የህክምና ቦታ ውስጥ በመስራቴ እሰራለሁ፡፡ | እዚህ የህክምና ቦታ ውስጥ በመስራቴ ኩራት ይሰማኛል። |
|  | Morale in this clinical area is high. | በዚህ የህክምና መስጫ አካባቢ ያለው የስራ ሞራል ከፍ ያለ ነው | በዚህ የህክምና ቦታ ላይ ሞራል ከፍተኛ ቦታ ይሰጠዋል፡፡ | በዚህ የህክምና ቦታ ያለው የስራ ተነሳሽነት እና በራስ መተማመን ከፍተኛ ነው፡፡ |
|  | When my workload becomes excessive, my performance is impaired. | የስራ ጫናዬ ሲበዛ፣ አፈፃፀሜ ይቀንሳል። | ከመጠን በላይ የስራ ጫና ሲበዛብኝ አፈጻጸሜ ይቀንሳል፡፡ | ከመጠን በላይ የስራ ጫና ሲበዛብኝ አፈጻጸሜ ይቀንሳል፡፡ |
|  | I am less effective at work when fatigued. | ሲደክመኝ በሥራ ላይ ውጤታማነቴ አነስተኛ ይሆናል | ድካም ውስጥ በምሆንበት ወቅት ውጤታማነቴ አነስተኛ ይሆናል፡፡. | ሲደክመኝ በሥራ ላይ ውጤታማነቴ አነስተኛ ይሆናል፡፡ |
|  | I am more likely to make errors in tense or hostile situations. | በውጥረት ወይም ጫና ባለበት ሁኔታዎች ውስጥ ስህተቶችን የመሥራት ዕድሌ ሰፊ ይሆናል | ውጥረት ወይም ምቾት የማይሰጡ ሁኔታዎች ባሉበት ስህተቶች የመስራት እድሌ ከፍተኛ ነው፡፡ | ውጥረት ወይም ጫና ባለበት ሁኔታዎች ውስጥ ስህተቶችን የመስራት እድሌ ከፍተኛ ነው፡፡ |
|  | Fatigue impairs my performance during emergency situations (e.g. emergency resuscitation, seizure). | ድካም ሲሰማኛ፤ ለድንገተኛ ህክምናዎች መስጠት ያለብኝ አገልግሎት ይጎዳል (e.g. emergency resuscitation, seizure) | ድካም በድንገተኛ/ አስቸኳይ ሁኔታዎች ወቅት (ምሳ፡ emergency resuscitation, seizure) አፈጻጸሜን ይቀንሰዋል፡፡ | ድካም ሲሰማኝ፤ ለድንገተኛ ህክምናዎች መስጠት ያለብኝ አገልግሎት ይቀንሳል፡፡(ምሳሌ፡ emergency resuscitation, seizure) |
|  | Management supports my daily efforts: | አስተዳደሩ የዕለት ተዕለት ጥረቴን ይደግፋል | የሚከተሉት አስተዳደር ለየዕለት ጥረቶቼ ድጋፎች ያደርግልኛል: | የሆስፒታሉ አስተዳደር(ማኔጅመንት) የዕለት ተዕለት ጥረቶቼን ይደግፋል፡፡ |
|  | Management doesn’t knowingly compromise pt safety: | የመስራያ ቤቴ ማኔጅመንት እያወቀ የታካሚውን ደህንነት አይጎዳውም | የሚከተሉት አስተዳደር ሆን ብሎ የታካሚ ደህንነትን ለአደጋ አያጋልጥም: | የሆስፒታሉ አስተዳደር (ማኔጅመንት) ሆን ብሎ (እያወቀ) የታካሚ ደህንነትን ለአደጋ አያጋልጥም፡ |
|  | Management is doing a good job: | አስተዳደሩ ጥሩ ስራ እየሰራ ነው፡ | የሚከተሉት አስተዳደር ጥሩ ስራ እየሰራ ነው: | የሆስፒታሉ አስተዳደር (ማኔጅመንት) ጥሩ ስራ እየሰራ ነው፡፡ |
|  | Problem personnel are dealt with constructively by our: | ችግሮች ከሰራተኞች ጋር ሲፈጠሩ አግባብ በሆነ መንገድ ነው የሚስተናገዱት | ችግር ያለባቸው ሰራተኞች በሚከተሉት ገንቢ በሆነ መልኩ ይስተናገዳሉ: | የሆስፒታሉ አስተዳደር (ማኔጅመንት) ችግር ያለባቸው ሰራተኞችን ገንቢ በሆነ መልኩ ያስተናገዳሉ: |
|  | I get adequate, timely info about events that might affect my work, from: | ስራዬን ሊነኩ ስለሚችሉ ክስተቶች በቂ እና ወቅታዊ መረጃ አገኛለሁ | ከሚከተሉት በስራ ሁኔታዬ ላይ ተጽእኖ ሊፈጥሩ የሚችሉ ሁኔታዎች የተመለከተ በቂና ወቅቱን የጠበቀ መረጃ አገኛለሁ፡ | በስራ ሁኔታዬ ላይ ተጽእኖ ሊፈጥሩ የሚችሉ ሁኔታዎች የተመለከተ በቂና ወቅቱን የጠበቀ መረጃ ከሆስፒታሉ አስተዳደር (ማኔጅመንት) አገኛለሁ፡ |
|  | The levels of staffing in this clinical area are sufficient to handle the number of patients. | በዚህ የህክምና መስጫ ያለው የሰራተኞች መጠን ከታካሚዎች አገልግሎት ለመስጠት በቂ ነው | በዚህ የህክምና ቦታ ላይ የሚደረገው የሰራተኛ ምደባ የሚመጡ ታካሚዎችን ለማስተናገድ በሚያስችል ልክ በቂ ነው፡፡ | በዚህ የህክምና ክፍል ውስጥ የሚደረገው የሰራተኛ ምደባ የሚመጡ ታካሚዎችን ለማስተናገድ በሚያስችል ልክ በቂ ነው፡፡ |
|  | This hospital does a good job of training new personnel. | ይህ ሆስፒታል አዳዲስ ሰራተኞችን በማሰልጠን ጥሩ ስራ እየሰራ ነው | ይህ ሆስፒታል አዲስ ሰራተኞችን በማስልጠን ረገድ ጥሩ ስራ እየሰራ ነው፡፡ | ይህ ሆስፒታል አዲስ ሰራተኞችን በማስልጠን ረገድ ጥሩ ስራ እየሰራ ነው፡፡ |
|  | All the necessary information for diagnostic and therapeutic decisions is routinely available to me. | ለምርመራ እና ለህክምና ውሳኔዎች የሚያስፈልጉኝን መረጃዎች በመደበኛነት አገኛለሁ | ሁሉም የምርመራና የህክምና ውሳኔዎች ለማሳለፍ የሚረዱ አስፈላጊ መረጃዎች በመደበኛነት ይቀርቡልኛል፡፡ | ሁሉንም የምርመራና የህክምና ውሳኔዎች ለማሳለፍ የሚረዱ አስፈላጊ መረጃዎች በመደበኛነትና ያለመቆራረጥ አገኛለሁ፡፡ |
|  | Trainees in my discipline are adequately supervised. | ሰልጣኞች በበቂ ሁኔታ ቁጥጥር ይደረግባቸዋል | በእኔ የትምህርት ዘርፍ ስር ያሉ ሰልጣኞች በበቂ ሁኔታ ቁጥጥር ይደረግላቸዋል፡፡ | በእኔ የስራ ሙያ ዘርፍ ስር የሚገኙ ሰልጣኞች በበቂ ሁኔታ ክትትልና ቁጥጥር ይደረግላቸዋል |
