# Supplementary figures and images for: Validation study of the Amharic version Safety Attitudes Questionnaire (SAQ) in public hospitals of Addis Ababa, Ethiopia: a cross-sectional study
Source: BMC Health Serv Res. 2024 Mar 22;24:366. doi: 10.1186/s12913-024-10865-9 (PMC10960426; doi:10.1186/s12913-024-10865-9)

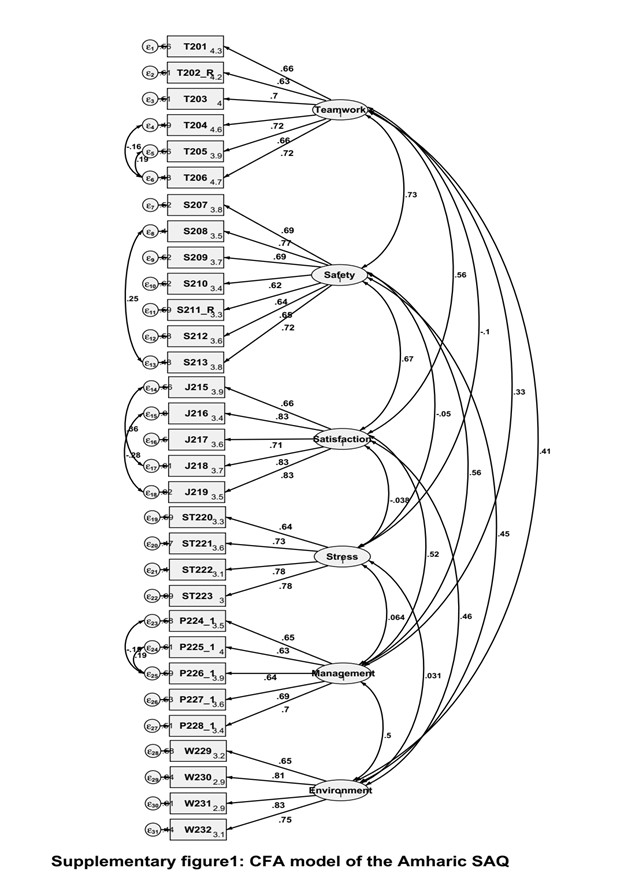

Supplement: Supplementary file 2 — Supplementary Material 2. [file 12913_2024_10865_MOESM2_ESM.jpg]
