# Supplementary material for: Validation study of the Amharic version Safety Attitudes Questionnaire (SAQ) in public hospitals of Addis Ababa, Ethiopia: a cross-sectional study
Source: BMC Health Serv Res. 2024 Mar 22;24:366. doi: 10.1186/s12913-024-10865-9 (PMC10960426; doi:10.1186/s12913-024-10865-9)
Supplement: Supplementary file 3 — Supplementary Material 3. [file 12913_2024_10865_MOESM3_ESM.docx]

Supplementary table 2: Factor Loadings of SAQ-A Observation Variables

| Factor | Item | Standardized Coefficient (λ) |
| --- | --- | --- |
| Teamwork Climate | Nurse input is well received in this clinical area. | 0.66 |
|  | In this clinical area, it is difficult to speak up if I perceive a problem with patient care. | 0.63 |
|  | Disagreements in this clinical area are resolved appropriately (i.e., not who is right, but what is best for the patient). | 0.70 |
|  | I have the support I need from other personnel to care for patients. | 0.72 |
|  | It is easy for personnel here to ask questions when there is something that they do not understand. | 0.66 |
|  | The physicians and nurses here work together as a well-coordinated team. | 0.72 |
| Safety Climate | I would feel safe being treated here as a patient. | 0.69 |
|  | Medical errors are handled appropriately in this clinical area. | 0.77 |
|  | I know the proper channels to direct questions regarding patient safety in this clinical area. | 0.69 |
|  | I receive appropriate feedback about my performance. | 0.62 |
|  | In this clinical area, it is difficult to discuss errors. | 0.64 |
|  | I am encouraged by my colleagues to report any patient safety concerns I may have. | 0.65 |
|  | The culture in this clinical area makes it easy to learn from the errors of others. | 0.72 |
| Job Satisfaction | I like my job. | 0.66 |
|  | Working here is like being part of a large family. | 0.83 |
|  | This is a good place to work. | 0.71 |
|  | I am proud to work in this clinical area. | 0.83 |
|  | Morale in this clinical area is high. | 0.83 |
|  | When my workload becomes excessive, my performance is impaired. | 0.64 |
|  | I am less effective at work when fatigued. | 0.73 |
|  | I am more likely to make errors in tense or hostile situations. | 0.78 |
|  | Fatigue impairs my performance during emergency situations (e.g., emergency resuscitation, seizure). | 0.78 |
| Perception of Management | Management supports my daily efforts: | 0.65 |
|  | Management doesn’t knowingly compromise patient safety: | 0.63 |
|  | Management is doing a good job: | 0.64 |
|  | Problem personnel are dealt with constructively by our: | 0.69 |
|  | I get adequate, timely info about events that might affect my work, from: | 0.70 |
| Work Environment | The levels of staffing in this clinical area are sufficient to handle the number of patients. | 0.65 |
|  | This hospital does a good job of training new personnel. | 0.81 |
|  | All the necessary information for diagnostic and therapeutic decisions is routinely available to me. | 0.83 |
|  | Trainees in my discipline are adequately supervised. | 0.75 |
